# Supplementary material for: Computational stabilization of T cell receptors allows pairing with antibodies to form bispecifics
Source: Nat Commun. 2020 May 11;11:2330. doi: 10.1038/s41467-020-16231-7 (PMC7214467; doi:10.1038/s41467-020-16231-7)
Supplement: Supplementary file 1 — Supplementary Information [file 41467_2020_16231_MOESM1_ESM.pdf]

Supplementary Information

Supplementary Table S1. Expression screening of Cα/Cβ subunits with mutations derived by modeling (single expression values).

Mutation (NY-ESO-1 peptide numbering)	Mutation (Kabat numbering)	Titer (mg/L)	Titer Ratio (Variant/ matched WT)	DSF T_m (°C)	Rosetta Predicted dE (REU)	Present in MSA	Region
Cα/Cβ wild-type[a,b]		12.8	1.00	54.9			
βP173A	βP178A	10.4	0.81		-1.61	+	Core
βE129K	βE134K	14.1	1.10	57.5	-2.34	+	Surface
αR126K	αR129K	17.5	1.37	53.1	-0.81	+	Interface
αV122L	αV125L	12.4	0.97		-1.17	+	Surface
βA123D	βA128D	10.4	0.81		-1.50	+	Interface
βK115S	βK120S	13.8	1.08	53.7	-1.56	+	Surface
αD183S	αD188S	16.1	1.26	53.3	0.66	+	Surface
αV176L	αV181L	11.3	0.88		-2.10	+	Interface
αT145I	αT150I	13.6	1.06	55.7	-1.66	+	Surface
βH134R	βH139R	15.0	1.17	57.1	-3.11	+	Interface
βQ199H	βQ204H	11.5	0.90		-3.11	+	Interface
βN116K	βN121K	5.3	0.41		-1.16	+	Surface
βE121T	βE126T	10.8	0.84		-1.30	+	Interface
αD154E	αD159E	12.2	0.95		-0.25	+	Surface
αA185T	αA190T	14.6	1.14	57.5	-1.87	+	Surface
αI157A	αI162A	8.6	0.67		3.61	-	Core
βL154A	βL159A	7.9	0.62		0.98	-	Core
βL143A	βL148A	1.7	0.13		1.74	+	Interface

Cα/Cβ wild-type		21.3	1.00	53.3			
αQ116K	αQ119K	34.2	1.61		-2.95	+	Surface
αR126F	αR129F	8.1	0.38		-3.45	-	Interface
αK129R	αK134R	30.9	1.45		-3.93	+	Interface
αS130G	αS135G	8.6	0.41		-5.85	-	Surface
αS131G	αS136G	35.7	1.68	52.7	-2.71	+	Surface
αS134F	αS139F	35.7	1.68	58.5	-4.50	-	Surface
αS134G	αS139G	28.4	1.33		0.92	-	Surface
αS134Y	αS139Y	23.0	1.08		-4.21	-	Surface
αS143D	αS148D	24.5	1.15		-4.19	-	Surface
αT145Q	αT150Q	22.7	1.07		-4.13	-	Surface
αK151V	αK156V	23.7	1.11		0.05	+	Surface
αK151A	αK156A	59.1	2.77	54.3	-2.76	+	Surface
αK151D	αK156D	26.6	1.25		-4.01	-	Surface
αK151N	αK156N	42.0	1.97	53.9	-3.57	-	Surface
αK151S	αK156S	26.9	1.26		-3.49	+	Surface
αS167Y	αS172Y	4.6	0.21		-4.05	-	Surface
αK171Y	αK176Y	4.7	0.22		-4.18	-	Surface
αN173Y	αN178Y	21.3	1.00		-3.13	+	Core
αN191Y	αN196Y	11.9	0.56		-3.86	-	Surface
βL114Y	βL119Y	28.9	1.35		-3.83	-	Core
βA123T	βA128T	39.0	1.83	53.9	-2.78	+	Interface
βA123V	βA128V	30.3	1.42		-2.99	+	Interface
βT135K	βT140K	6.5	0.30		-2.76	+	Interface

βQ136G	βQ141G	4.7	0.22		-3.26	+	Surface
βK137L	βK142L	26.2	1.23		-4.52	-	Interface
βD150P	βD155P	47.4	2.23	61.2	-3.72	-	Interface /Core
βV158I	βV163I	27.7	1.30		-4.00	-	Surface
βS165D	βS170D	58.8	2.76	58.5	-2.84	+	Interface
βS165E	βS170E	37.8	1.77	57.5	-4.12	-	Interface
βD170Q	βD175Q	26.4	1.23		-3.92	+	Interface
βA179W	βQ184W	18.6	0.87		-3.49	-	Surface
βA179Y	βQ184Y	4.8	0.23		-3.44	-	Surface
βS194H	βS199H	26.0	1.22		-3.69	-	Surface
βK137T	βK142T	20.9	0.98		-4.06	+	Interface

[a]All CαCβ fragments contain the αT166C/βS173C disulfide.
[b]The screen was split into two groups, which are separated by the two CαCβ wild-type controls.

Supplementary Table S2

**Data Collection**	
Space group	C2
Cell dimensions	
a, b, c (Å)	96.85, 59.85, 61.35
α, β, γ (°)	90, 110.2, 90
Resolution (Å)	29.33–1.76 (1.86–1.76)*
R_{merge}	0.057 (0.910)*
I/σI	5.6 (0.8)*
$CC_{1/2}$	0.999 (0.588)
Completeness (%)	99.2 (99.1)*
Redundancy	3.8 (3.6)*

*Values in parentheses are for the highest resolution shell.

**Refinement**	
No. of reflections	30,717

R_{free}	0.230
R_{work}	0.209
No. of amino acids	217
No. of atoms	
Protein	1726
Ligand/ion	3
Water	175
B-factors	
Protein	30.1
Ligand/ion	29.5
Water	37.4
RMS deviations	
Bond lengths (Å)	0.007
Bond angles (°)	1.242
Ramachandran plot (%)	
Favored	99.5
Allowed	0.5
Outliers	0.0

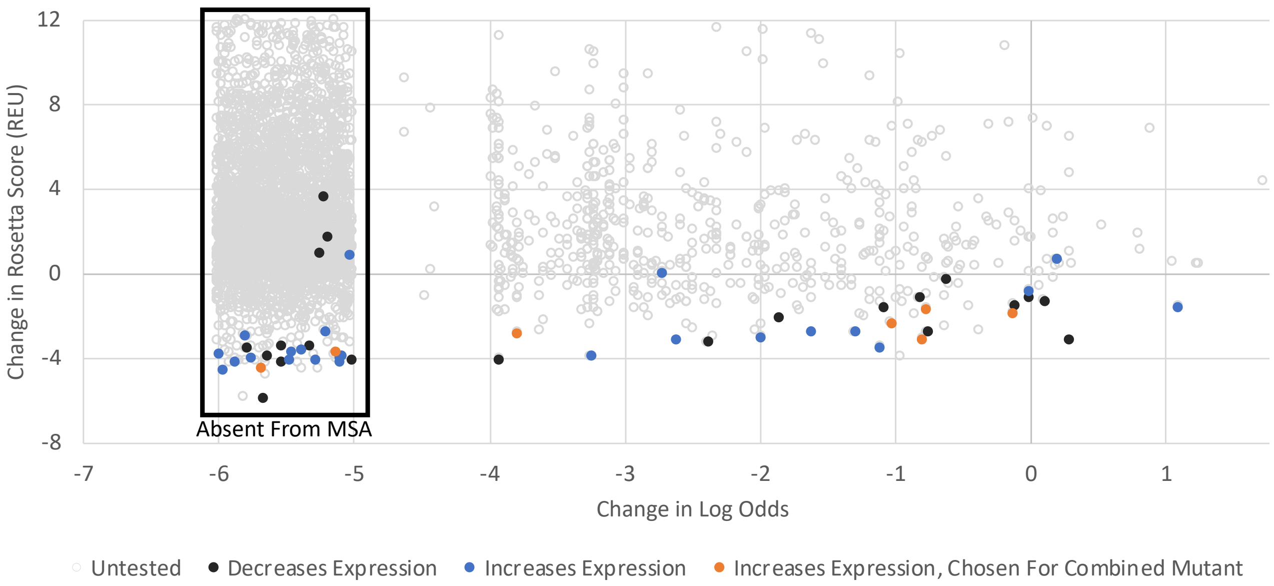

Supplementary Figure S1. Computational metrics for all possible single point mutations. Each mutation was measured by log odds score derived from our MSA (x-axis, more positive is favorable) and Rosetta score (y-axis, more negative is favorable). Mutations absent from the MSA are shown on the left, given random x-axis values for the purpose of visualization. Each mutation is classified as one of four labels. Single point mutations shown in black resulted in lower expression levels when tested experimentally. Conversely, mutations shown in blue resulted in equal or higher expression levels. The seven mutations chosen for our combination are shown in orange, each of which had expression levels higher than wild type. Mutations that were not experimentally tested are shown in gray.

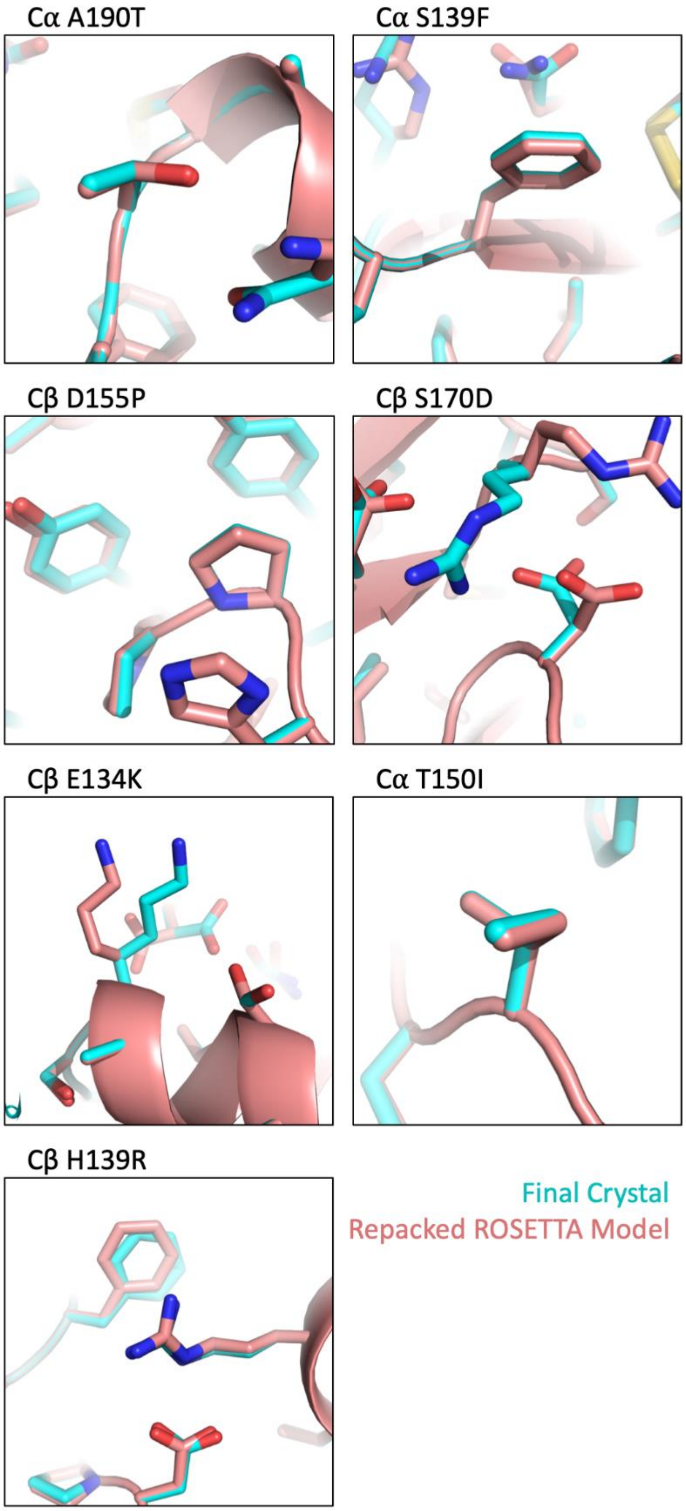

Supplementary Figure S2. Comparison of the crystal structure of our 7-mutant TCR (cyan) and the output of Rosetta when it is used to predict the conformations of the amino acid side chains given the backbone coordinates of the 7-mutant (brown).

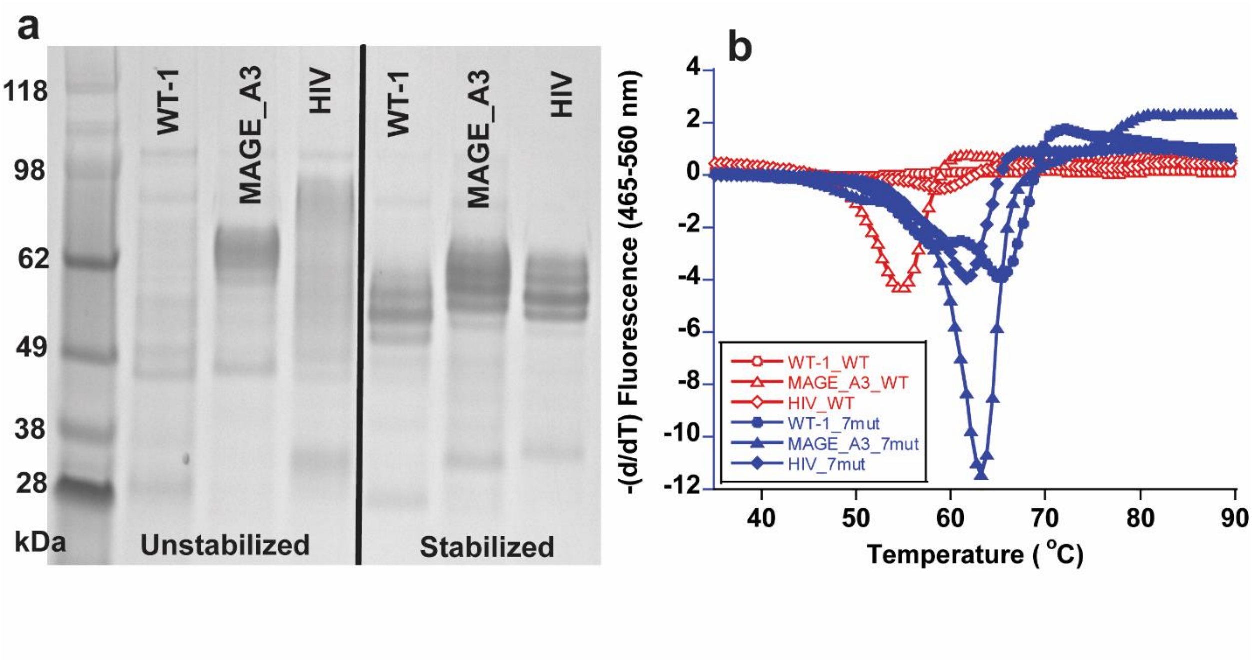

Supplementary Figure S3. Characterization of 3 additional TCR with and without the Cα/Cβ stabilizing designs (all without Cα166/Cβ173 disulfide). (**a**) SDS-PAGE analysis of the assembly of each of the purified TCRs. Clearly the non-stabilized Wilm's Tumor-1 (WT-1) and HIV TCRs lack distinct bands expected for a properly assembled α/β TCR, while all three of the TCRs demonstrate properly assembled α/β TCR bands (glycosylation leads to multiple bands). (**b**) Differential scanning fluorimetry curves of the non-stabilized (WT, red) and stabilized (7mut, blue) α/β TCRs. All points on the DSF plots are single measured fluorescence values at each temperature.

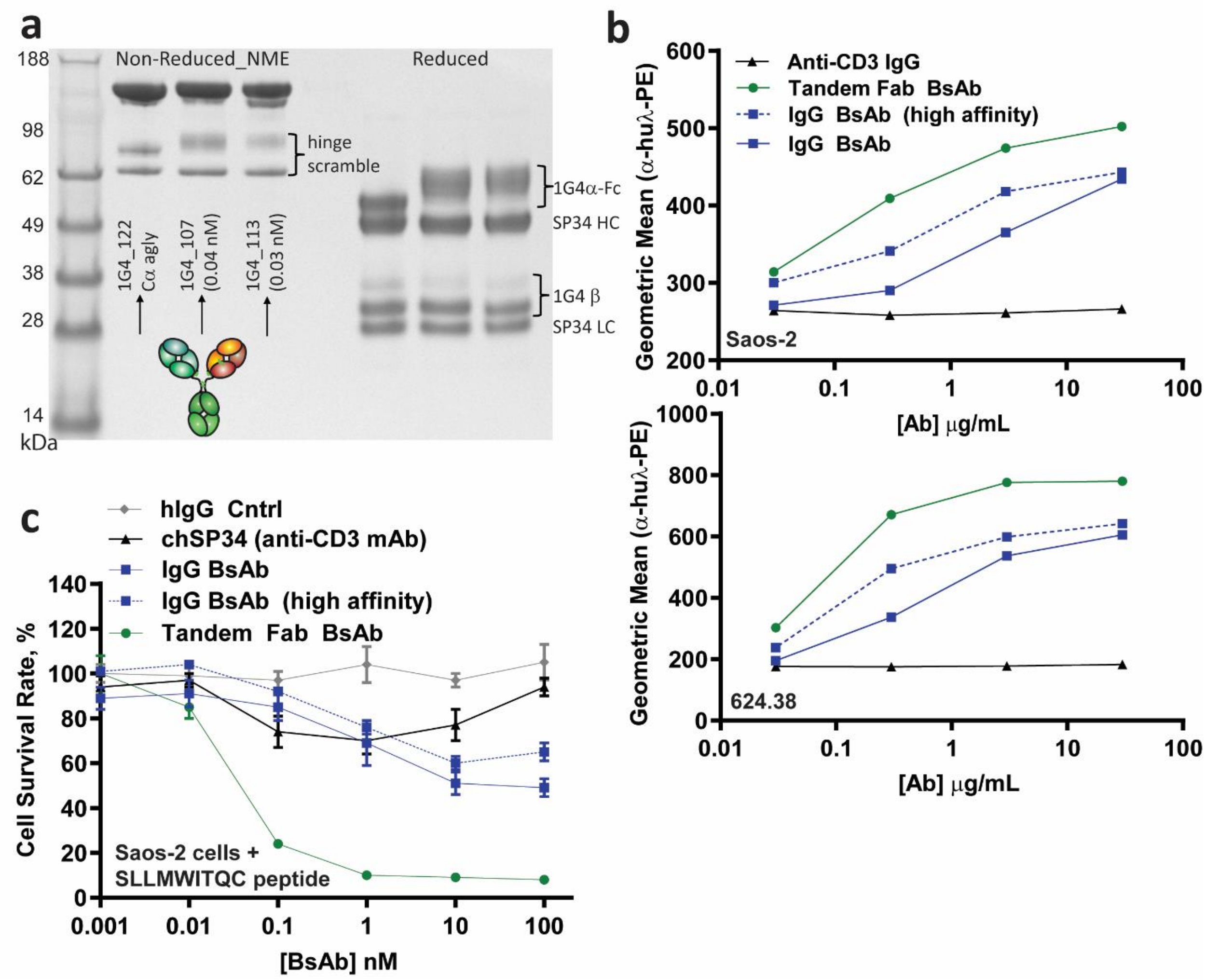

Supplementary Figure S4. Additional TCR/CD3 IgG bifunctionals with Cα glycosylation knocked-out or with higher affinity to HLA-A2/NY-ESO-1 peptide. **a** Reduced and non-reduced SDS-PAGE of the IgG BsAbs with different affinity or with Cα deglycosylated by mutation of the canonical N-linked glycosylation sites. **b** Flow cytometry cell binding titrations of the BsAb molecules onto Saos-2 (top) and 624.38 (middle) cells pulsed with the NY-ESO-1 peptide SLLMWITQC. Each data point is the Geometric mean of the fluorescence of the cells labeled with the CD3 mAb or TCR/CD3 BsAb and the secondary antibody. **c** T cell redirected lysis activity of Saos-2 sarcoma cells by an affinity matured TCR/CD3 IgG BsAb and comparative controls. For **c**, the data points are the mean of 3 replicates and the error bars are the standard deviation.

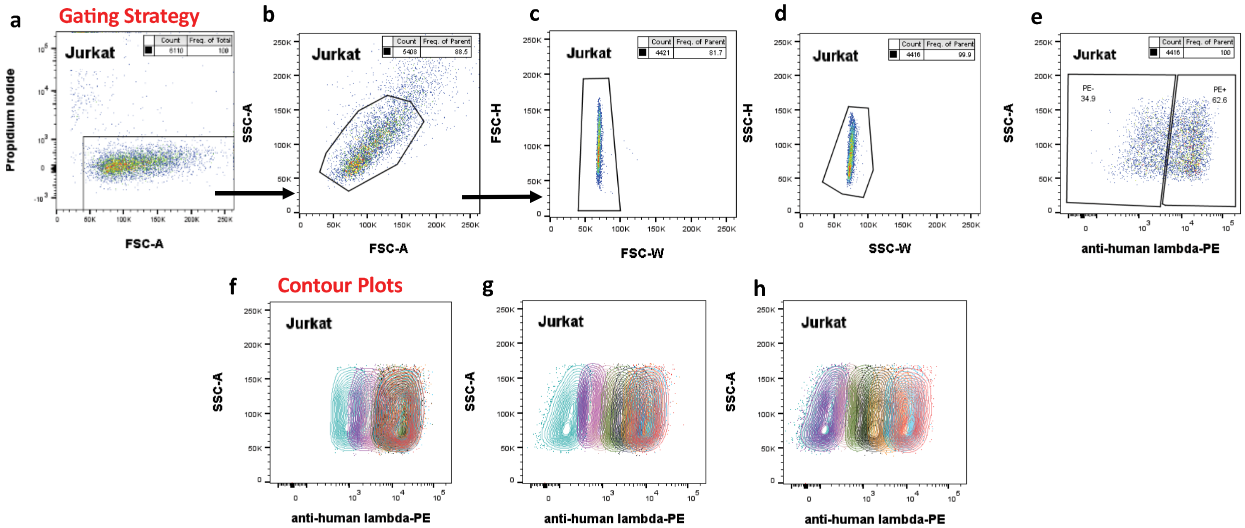

	WELL ID	Count	Geometric Mean : Comp-PE-A
	A01	2766	15309
	A02	2930	14974
	A03	2911	14572
	A04	2753	13348
	A05	2545	10474
	A06	2525	5528
	A07	2221	2620
	A08	2326	1022

	WELL ID	Count	Geometric Mean : Comp-PE-A
	B01	2354	10408
	B02	2242	9750
	B03	2328	7714
	B04	2450	4625
	B05	2187	2351
	B06	1730	1073
	B07	1303	558
	B08	4426	184

	WELL ID	Count	Geometric Mean : Comp-PE-A
	C01	2280	11628
	C02	2185	8415
	C03	2480	3889
	C04	2286	1831
	C05	1683	910
	C06	1135	472
	C07	4338	168
	C08	4373	144

Supplementary Figure S5. Flow cytometry gating strategy for detecting TCR/CD3 BsAb binding to Jurkat cells (Figure 7a – right panel). Cells were initially gated based on **(a)** FSC-A vs Propidium Iodide to exclude dead cells. **(b)** FSC-A vs SSC-A to identify the distribution of cells based on light scatter. **(c** and **d)** FSC-W vs FSC-H and SSC-W vs SSC-H for doublet discrimination. (**e**) anti-human lambda-PE vs SSC-A for determination of positivity. Contour Plots: 8-point titrations starting at 30 mg/mL (1:3 dilutions) of chimeric SP34 anti-CD3 **(f),** TCR/CD3 IgG BsAb **(g),** and TCR/CD3 Tandem Fab BsAb **(h)** onto Jurkat cells.

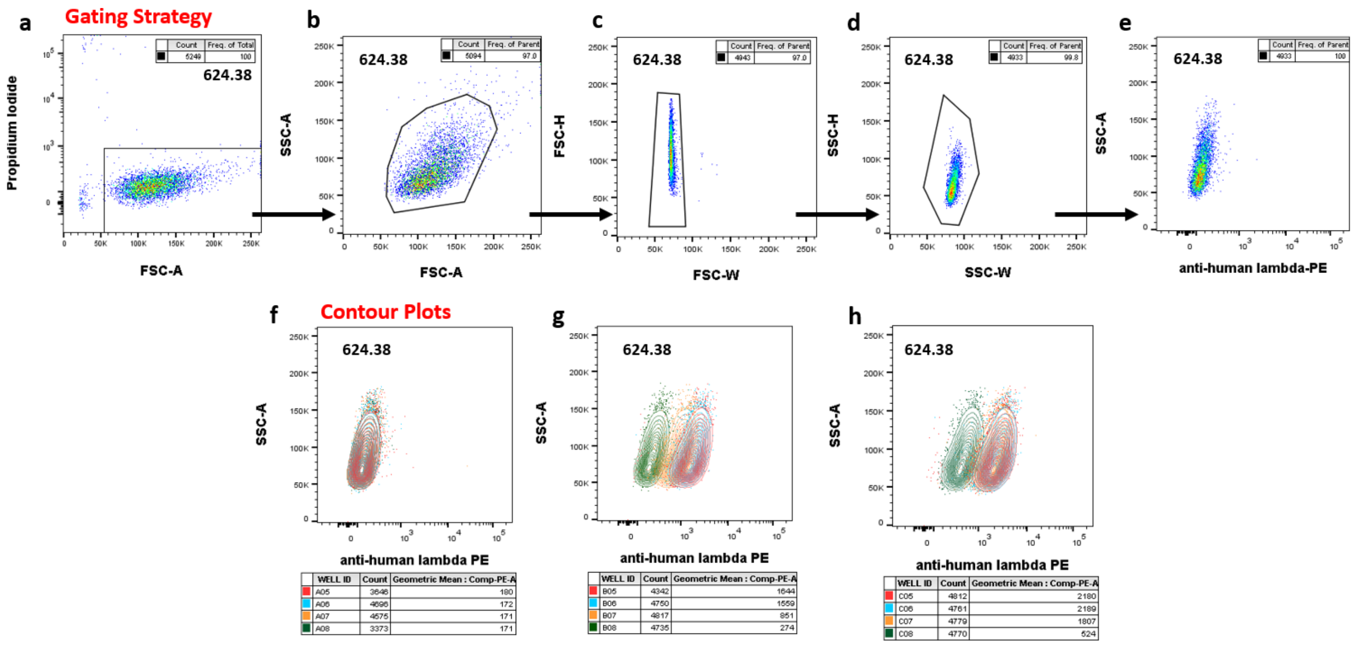

Supplementary Figure S6. Flow cytometry gating strategy for detecting TCR/CD3 BsAb binding to 624.38 cells. Cells were initially gated based on **(a)** FSC-A vs Propidium Iodide to exclude dead cells. **(b)** FSC-A vs SSC-A to identify the distribution of cells based on light scatter. **(c** and **d)** FSC-W vs FSC-H and SSC-W vs SSC-H for doublet discrimination. (**e**) anti-human lambda-PE vs SSC-A for determination of positivity. Contour Plots: 30, 3, 0.3, and 0.03 µg/mL titration of chimeric SP34 anti-CD3 **(f),** TCR/CD3 IgG BsAb **(g),** and TCR/CD3 Tandem Fab BsAb **(h)** onto 628.34 cells.

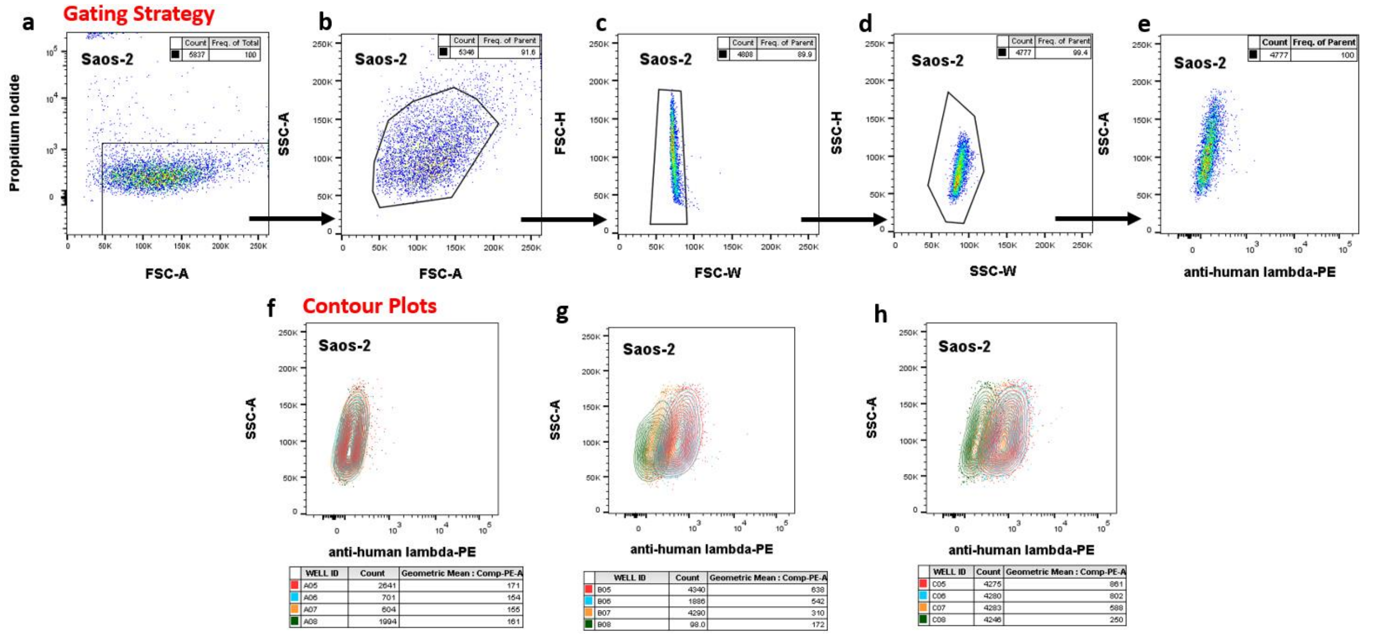

WELL ID	Count	Geometric Mean : Comp-PE-A
A05	2641	171
A06	701	154
A07	604	155
A08	1994	161

WELL ID	Count	Geometric Mean : Comp-PE-A
B05	4340	638
B06	1886	542
B07	4290	310
B08	98.0	172

WELL ID	Count	Geometric Mean : Comp-PE-A
C05	4275	861
C06	4280	802
C07	4283	588
C08	4246	250

Supplementary Figure S7. Flow cytometry gating strategy for detecting TCR/CD3 BsAb binding to Saos-2 cells (Figure 7a – left panel). Cells were initially gated based on **(a)** FSC-A vs Propidium Iodide to exclude dead cells. **(b)** FSC-A vs SSC-A to identify the distribution of cells based on light scatter. **(c** and **d)** FSC-W vs FSC-H and SSC-W vs SSC-H for doublet discrimination. (**e**) anti-human lambda-PE vs SSC-A for determination of positivity. Contour Plots: 30, 3, 0.3, and 0.03 μg/mL titration of chimeric SP34 anti-CD3 **(f),** TCR/CD3 IgG BsAb **(g),** and TCR/CD3 Tandem Fab BsAb **(h)** onto Saos-2 cells.

Script for running Rosetta simulations for single point mutants. Each mutation is defined in a “resfile” (see below)

```
<ROSETTASCRIPTS>
  <TASKOPERATIONS>
    <InitializeFromCommandline name="ifc"/>
    <ReadResfile name="rrf" filename="resfile"/>
  </TASKOPERATIONS>

  <SCOREFXNS>
    <ScoreFunction name="beta_cart_sfxn" weights="beta_nov15_cart"/>

    <ScoreFunction name="beta_cart_sfxn_cc" weights="beta_nov15_cart">
      <Reweight scoretype="coordinate_constraint" weight="1"/>
    </ScoreFunction>
  </SCOREFXNS>

  <MOVERS>
    <AtomCoordinateCstMover name="coor_cst"/>
    <FastRelax name="fast_relax" scorefxn="beta_cart_sfxn_cc" repeats="5"
cartesian="true" task_operations="ifc,rrf"/>
    <MinMover name="min_mover" scorefxn="beta_cart_sfxn" chi="true" bb="true" cartesian="true"/>
  </MOVERS>

  <PROTOCOLS>
    <Add mover="coor_cst"/>
    <Add mover="fast_relax"/>
    <Add mover="min_mover"/>
  </PROTOCOLS>

  <OUTPUT scorefxn="beta_cart_sfxn"/>
</ROSETTASCRIPTS>
```

Example resfile to be fed into the Rosetta script. This example mutates residue 114 of chain D (alpha chain) to Glutamic Acid, allows all neighbors (residue positions listed below "start" line, selected based on distance to mutation) to sample alternative side chain rotamers without mutating, and fixes all other positions to their current side chain rotamer. The control for this mutation can be measured by replacing "PIKAA E" with "NATAA" on the third line of the script.

```
NATRO
start
114 D PIKAA E USE_INPUT_SC EX 1 LEVEL 1 EX 2 LEVEL 1 EX 3 LEVEL 1 EX 4 LEVEL 1
115 D NATAA USE_INPUT_SC EX 1 LEVEL 1 EX 2 LEVEL 1 EX 3 LEVEL 1 EX 4 LEVEL 1
116 D NATAA USE_INPUT_SC EX 1 LEVEL 1 EX 2 LEVEL 1 EX 3 LEVEL 1 EX 4 LEVEL 1
117 D NATAA USE_INPUT_SC EX 1 LEVEL 1 EX 2 LEVEL 1 EX 3 LEVEL 1 EX 4 LEVEL 1
118 D NATAA USE_INPUT_SC EX 1 LEVEL 1 EX 2 LEVEL 1 EX 3 LEVEL 1 EX 4 LEVEL 1
119 D NATAA USE_INPUT_SC EX 1 LEVEL 1 EX 2 LEVEL 1 EX 3 LEVEL 1 EX 4 LEVEL 1
120 D NATAA USE_INPUT_SC EX 1 LEVEL 1 EX 2 LEVEL 1 EX 3 LEVEL 1 EX 4 LEVEL 1
121 D NATAA USE_INPUT_SC EX 1 LEVEL 1 EX 2 LEVEL 1 EX 3 LEVEL 1 EX 4 LEVEL 1
138 D NATAA USE_INPUT_SC EX 1 LEVEL 1 EX 2 LEVEL 1 EX 3 LEVEL 1 EX 4 LEVEL 1
139 D NATAA USE_INPUT_SC EX 1 LEVEL 1 EX 2 LEVEL 1 EX 3 LEVEL 1 EX 4 LEVEL 1
140 D NATAA USE_INPUT_SC EX 1 LEVEL 1 EX 2 LEVEL 1 EX 3 LEVEL 1 EX 4 LEVEL 1
141 D NATAA USE_INPUT_SC EX 1 LEVEL 1 EX 2 LEVEL 1 EX 3 LEVEL 1 EX 4 LEVEL 1
142 D NATAA USE_INPUT_SC EX 1 LEVEL 1 EX 2 LEVEL 1 EX 3 LEVEL 1 EX 4 LEVEL 1
143 D NATAA USE_INPUT_SC EX 1 LEVEL 1 EX 2 LEVEL 1 EX 3 LEVEL 1 EX 4 LEVEL 1
144 D NATAA USE_INPUT_SC EX 1 LEVEL 1 EX 2 LEVEL 1 EX 3 LEVEL 1 EX 4 LEVEL 1
145 D NATAA USE_INPUT_SC EX 1 LEVEL 1 EX 2 LEVEL 1 EX 3 LEVEL 1 EX 4 LEVEL 1
146 D NATAA USE_INPUT_SC EX 1 LEVEL 1 EX 2 LEVEL 1 EX 3 LEVEL 1 EX 4 LEVEL 1
147 D NATAA USE_INPUT_SC EX 1 LEVEL 1 EX 2 LEVEL 1 EX 3 LEVEL 1 EX 4 LEVEL 1
159 D NATAA USE_INPUT_SC EX 1 LEVEL 1 EX 2 LEVEL 1 EX 3 LEVEL 1 EX 4 LEVEL 1
160 D NATAA USE_INPUT_SC EX 1 LEVEL 1 EX 2 LEVEL 1 EX 3 LEVEL 1 EX 4 LEVEL 1
161 D NATAA USE_INPUT_SC EX 1 LEVEL 1 EX 2 LEVEL 1 EX 3 LEVEL 1 EX 4 LEVEL 1
162 D NATAA USE_INPUT_SC EX 1 LEVEL 1 EX 2 LEVEL 1 EX 3 LEVEL 1 EX 4 LEVEL 1
163 D NATAA USE_INPUT_SC EX 1 LEVEL 1 EX 2 LEVEL 1 EX 3 LEVEL 1 EX 4 LEVEL 1
164 D NATAA USE_INPUT_SC EX 1 LEVEL 1 EX 2 LEVEL 1 EX 3 LEVEL 1 EX 4 LEVEL 1
165 D NATAA USE_INPUT_SC EX 1 LEVEL 1 EX 2 LEVEL 1 EX 3 LEVEL 1 EX 4 LEVEL 1
166 D NATAA USE_INPUT_SC EX 1 LEVEL 1 EX 2 LEVEL 1 EX 3 LEVEL 1 EX 4 LEVEL 1
167 D NATAA USE_INPUT_SC EX 1 LEVEL 1 EX 2 LEVEL 1 EX 3 LEVEL 1 EX 4 LEVEL 1
168 D NATAA USE_INPUT_SC EX 1 LEVEL 1 EX 2 LEVEL 1 EX 3 LEVEL 1 EX 4 LEVEL 1
169 D NATAA USE_INPUT_SC EX 1 LEVEL 1 EX 2 LEVEL 1 EX 3 LEVEL 1 EX 4 LEVEL 1
170 D NATAA USE_INPUT_SC EX 1 LEVEL 1 EX 2 LEVEL 1 EX 3 LEVEL 1 EX 4 LEVEL 1
171 D NATAA USE_INPUT_SC EX 1 LEVEL 1 EX 2 LEVEL 1 EX 3 LEVEL 1 EX 4 LEVEL 1
172 D NATAA USE_INPUT_SC EX 1 LEVEL 1 EX 2 LEVEL 1 EX 3 LEVEL 1 EX 4 LEVEL 1
173 D NATAA USE_INPUT_SC EX 1 LEVEL 1 EX 2 LEVEL 1 EX 3 LEVEL 1 EX 4 LEVEL 1
174 D NATAA USE_INPUT_SC EX 1 LEVEL 1 EX 2 LEVEL 1 EX 3 LEVEL 1 EX 4 LEVEL 1
175 D NATAA USE_INPUT_SC EX 1 LEVEL 1 EX 2 LEVEL 1 EX 3 LEVEL 1 EX 4 LEVEL 1
135 E NATAA USE_INPUT_SC EX 1 LEVEL 1 EX 2 LEVEL 1 EX 3 LEVEL 1 EX 4 LEVEL 1
137 E NATAA USE_INPUT_SC EX 1 LEVEL 1 EX 2 LEVEL 1 EX 3 LEVEL 1 EX 4 LEVEL 1
139 E NATAA USE_INPUT_SC EX 1 LEVEL 1 EX 2 LEVEL 1 EX 3 LEVEL 1 EX 4 LEVEL 1
165 E NATAA USE_INPUT_SC EX 1 LEVEL 1 EX 2 LEVEL 1 EX 3 LEVEL 1 EX 4 LEVEL 1
166 E NATAA USE_INPUT_SC EX 1 LEVEL 1 EX 2 LEVEL 1 EX 3 LEVEL 1 EX 4 LEVEL 1
167 E NATAA USE_INPUT_SC EX 1 LEVEL 1 EX 2 LEVEL 1 EX 3 LEVEL 1 EX 4 LEVEL 1
168 E NATAA USE_INPUT_SC EX 1 LEVEL 1 EX 2 LEVEL 1 EX 3 LEVEL 1 EX 4 LEVEL 1
171 E NATAA USE_INPUT_SC EX 1 LEVEL 1 EX 2 LEVEL 1 EX 3 LEVEL 1 EX 4 LEVEL 1
190 E NATAA USE_INPUT_SC EX 1 LEVEL 1 EX 2 LEVEL 1 EX 3 LEVEL 1 EX 4 LEVEL 1
192 E NATAA USE_INPUT_SC EX 1 LEVEL 1 EX 2 LEVEL 1 EX 3 LEVEL 1 EX 4 LEVEL 1
```

Example command line call:

```
rosetta_scripts.default.linuxgccrelease -parser:protocol run.xml -resfile resfile.txt -s my_protein.pdb
```

(where run.xml is shown on the previous page and resfile.txt is shown on this page)

Rosetta c++ snippet used to create resfiles:

```
std::string get_line_for_resid( char chain, core::Size resnum, char aa = 'x' ){
        std::stringstream ss;
        ss << resnum << " " << chain;
        if( aa == 'x' ) ss << " NATAA";
        else ss << " PIKAA " << aa;
        ss << " USE_INPUT_SC EX 1 LEVEL 1 EX 2 LEVEL 1 EX 3 LEVEL 1 EX 4 LEVEL 1";
        return ss.str();
}

void make_resfile( std::string const & filename, core::Size resid, char aa, core::pose::Pose & pose,
utility::graph::GraphOP packer_neighbor_graph ){
        std::ofstream outfile;
        outfile.open( filename );

        outfile << "NATRO" << std::endl << "start" << std::endl;
        outfile << get_line_for_resid( pose.pdb_info()->chain( resid ), pose.pdb_info()->number( resid ),
aa ) << std::endl;

        utility::graph::Node * node = packer_neighbor_graph->get_node( resid );

        for( utility::graph::EdgeListConstIterator it = node->const_edge_list_begin();
                         it != node->const_edge_list_end(); ++it){
                core::Size const other_resid = (*it)->get_other_ind( resid );
                if( other_resid != resid ){
                        outfile << get_line_for_resid( info( pose.pdb_info()->chain( other_resid ),
pose.pdb_info()->number( other_resid ) ) ) << std::endl;
                }
        }

        outfile.close();
}
```

Script for relaxing crystal structures:

```
<ROSETTASCRIPTS>
  <TASKOPERATIONS>
    <InitializeFromCommandline name="ifc"/> <!-- pass -ex1 -ex2 -->
  </TASKOPERATIONS>


  <SCOREFXNS>
    <ScoreFunction name="beta_sfxn_cc" weights="beta_nov15">
      <Reweight scoretype="coordinate_constraint" weight="1"/>
    </ScoreFunction>
  </SCOREFXNS>


  <MOVERS>
    <AtomCoordinateCstMover name="coor_cst"/>
    <FastRelax name="fast_relax" scorefxn="beta_sfxn_cc" repeats="5" task_operations="ifc"/>
  </MOVERS>


  <PROTOCOLS>
    <Add mover="coor_cst"/>
    <Add mover="fast_relax"/>
  </PROTOCOLS>


  <OUTPUT scorefxn="commandline"/>
</ROSETTASCRIPTS>
```
