# Supplementary material for: Computational stabilization of T cell receptors allows pairing with antibodies to form bispecifics
Source: Nat Commun. 2020 May 11;11:2330. doi: 10.1038/s41467-020-16231-7 (PMC7214467; doi:10.1038/s41467-020-16231-7)
Supplement: Supplementary file 3 — Reporting Summary [file 41467_2020_16231_MOESM3_ESM.pdf]

nature research

Corresponding author(s): Brian Kuhlman and Stephen Demarest

Last updated by author(s): Apr 4, 2020

Reporting Summary

Nature Research wishes to improve the reproducibility of the work that we publish. This form provides structure for consistency and transparency in reporting. For further information on Nature Research policies, see Authors & Referees and the Editorial Policy Checklist .

Statistics

For all statistical analyses, confirm that the following items are present in the figure legend, table legend, main text, or Methods section.

n/a	Confirmed	
☐	☒	The exact sample size (*n*) for each experimental group/condition, given as a discrete number and unit of measurement
☐	☒	A statement on whether measurements were taken from distinct samples or whether the same sample was measured repeatedly
☐	☒	The statistical test(s) used AND whether they are one- or two-sided<br>*Only common tests should be described solely by name; describe more complex techniques in the Methods section.*
☒	☐	A description of all covariates tested
☒	☐	A description of any assumptions or corrections, such as tests of normality and adjustment for multiple comparisons
☒	☐	A full description of the statistical parameters including central tendency (e.g. means) or other basic estimates (e.g. regression coefficient) AND variation (e.g. standard deviation) or associated estimates of uncertainty (e.g. confidence intervals)
☐	☒	For null hypothesis testing, the test statistic (e.g. *F*, *t*, *r*) with confidence intervals, effect sizes, degrees of freedom and *P* value noted<br>*Give P values as exact values whenever suitable.*
☒	☐	For Bayesian analysis, information on the choice of priors and Markov chain Monte Carlo settings
☒	☐	For hierarchical and complex designs, identification of the appropriate level for tests and full reporting of outcomes
☒	☐	Estimates of effect sizes (e.g. Cohen's *d*, Pearson's *r*), indicating how they were calculated

Our web collection on statistics for biologists contains articles on many of the points above.

Software and code

Policy information about availability of computer code

Data collection	The software for the XRAY data collection and analyses was referenced in the methods. The exact version numbers are listed in the pdb validation report, which is publicly available at RCSB.org as part of the pdb submission.
Data analysis	The versions of the Rosetta suite used for design were described in the methods and scripts for running the analyses are provided in the supplementary materials. Access to the exact scripts used within Rosetta have been provided in the Data Availability section.

For manuscripts utilizing custom algorithms or software that are central to the research but not yet described in published literature, software must be made available to editors/reviewers. We strongly encourage code deposition in a community repository (e.g. GitHub). See the Nature Research guidelines for submitting code & software for further information.

Data

Policy information about availability of data

All manuscripts must include a data availability statement. This statement should provide the following information, where applicable:

- Accession codes, unique identifiers, or web links for publicly available datasets
- A list of figures that have associated raw data
- A description of any restrictions on data availability

Data availability

The coordinates for the TCR constant domain structure, and the corresponding structure factors, have been deposited in the Protein Data Bank (http://www.rcsb.org) under accession code 6U07. Custom Scripts for running computational single residue mutations in Rosetta are provided in the Supplementary Information as well as in the Rosetta Commons: rosetta_scripts_scripts/scripts/public/point_mutant_scan/point_mutant_scan.Froning_et_al.xml. To run the scripts, users must have access to Rosetta software (https://www.rosettacommons.org/software/license-and-download). The scripts can also be found at https://gist.github.com/JackMaguire/6f33119ff9cc4e46e16dc860cdf306e2. Raw data for the figures described in the manuscript are available upon request.

Field-specific reporting

Please select the one below that is the best fit for your research. If you are not sure, read the appropriate sections before making your selection.

- [x] Life sciences
- [ ] Behavioural & social sciences
- [ ] Ecological, evolutionary & environmental sciences

For a reference copy of the document with all sections, see nature.com/documents/nr-reporting-summary-flat.pdf

Life sciences study design

All studies must disclose on these points even when the disclosure is negative.

Sample size	No in vivo models were included in the report. Much of the biochemical data consists of SDS-PAGE gels, chromatograms, and DSF thermograms, which are broad measures of our samples' properties where replicates are not necessary. For cell killing studies, all points are the mean of 3 replicates and the error bars are the standard deviation from the mean. This information is provided in the figure legend.
Data exclusions	No data points were excluded from the studies.
Replication	Were applicable, discussions of replication of data within each of the tables and figures is provided in the Table or Figure legends.
Randomization	No randomization was required as there were no in vivo or clinical studies in the report.
Blinding	No blinding equired as there were no in vivo or clinical studies in the report.

Reporting for specific materials, systems and methods

We require information from authors about some types of materials, experimental systems and methods used in many studies. Here, indicate whether each material, system or method listed is relevant to your study. If you are not sure if a list item applies to your research, read the appropriate section before selecting a response.

Materials & experimental systems

n/a	Involved in the study	
[ ]	[x]	Antibodies
[ ]	[x]	Eukaryotic cell lines
[x]	[ ]	Palaeontology
[x]	[ ]	Animals and other organisms
[x]	[ ]	Human research participants
[x]	[ ]	Clinical data

Methods

n/a	Involved in the study	
[x]	[ ]	ChIP-seq
[ ]	[x]	Flow cytometry
[x]	[ ]	MRI-based neuroimaging

Antibodies

Antibodies used	The source and human lambda light chain selectivity of the labeled commercial polyclonal antibody used for flow cytometry is provided along with their catalog/lot numbers and dilutions in the methods section. The primary antibody (anti-CD3) used in the study was generated recombinantly in-house and derived from SP34. We have published previously on this mAb and provide a reference. The control IgG used in the cell killing studies was an anti-CD20 mAb (not expressed on T cells or tumor cells) and a reference was provided describing its activity.
Validation	BD Biosciences' validation of the polyclonal 2ndary antibody we used for flow cytometry included demonstration of its ability to stain lambda LC-containing primary B lymphocytes from a human donor along with the remaining primary B lymphocytes showing staining by their goat anti-kappa LC polyclonal. The primary antibodies were all in-house produced antibodies (including the anti-CD3) and references were provided in the methods section describing their previous use/validation.

Eukaryotic cell lines

Policy information about cell lines

Cell line source(s)	(Directly from the methods): Saos-2 and A375 cells were from ATCC (Cat#HTB-85 and CRL-1619, respectively) and the 624.38 cells were from the NCI/NIH DTP, DCTD Tumor Repository (ref 58 - Describes the origin of the cell line)
Authentication	We did not authenticate the purchased tumor cell lines (Saos-2, 624.38, or A375).
Mycoplasma contamination	All cell lines were confirmed mycoplasma negative.

Commonly misidentified lines (See ICLAC register)

None

Flow Cytometry

Plots

Confirm that:

- [x] The axis labels state the marker and fluorochrome used (e.g. CD4-FITC).
- [x] The axis scales are clearly visible. Include numbers along axes only for bottom left plot of group (a 'group' is an analysis of identical markers).
- [x] All plots are contour plots with outliers or pseudocolor plots.
- [x] A numerical value for number of cells or percentage (with statistics) is provided.

Methodology

Sample preparation

Flow cytometry was only performed on cell lines. No flow cytometry was performed on primary cells.

Instrument

Becton Dickinson LSRFortessa flow cytometer. (as stated in the methods)

Software

BD FACSDiva software (v8.0.1) was used to run the cytometer and data was analyzed using FlowJo version 10.5.3.(as stated in the methods)

Cell population abundance

Cell line abundance is listed in the Supplementary FACS plots describing the gating strategies.

Gating strategy

The exact gating strategies have been exemplified in three supplementary figures for all three cell lines analyzed using flow cytometry.

- [x] Tick this box to confirm that a figure exemplifying the gating strategy is provided in the Supplementary Information.
