# Supplementary material for: Computational stabilization of T cell receptors allows pairing with antibodies to form bispecifics
Source: Nat Commun. 2020 May 11;11:2330. doi: 10.1038/s41467-020-16231-7 (PMC7214467; doi:10.1038/s41467-020-16231-7)
Supplement: Supplementary file 4 — Description of Additional Supplementary Files [file 41467_2020_16231_MOESM4_ESM.pdf]

Title: Supplementary Data 1

Description: This file contains the DNA and amino acid sequences of all the constructs and proteins characterized in the report.
